# The Risk of Ischemic Stroke in Patients with Chronic Obstructive Pulmonary Disease and Atrial Fibrillation

**DOI:** 10.3390/life15020154

**Published:** 2025-01-22

**Authors:** Hsien-Lung Tsai, Chih-Chun Hsiao, Yu-Hsuan Chen, Wu-Chien Chien, Chi-Hsiang Chung, Chun-Gu Cheng, Chun-An Cheng

**Affiliations:** 1 Department of Emergency Medicine, Cheng Hsin General Hospital, Taipei 11220, Taiwan; ufshanno@ms15.hinet.net; 2 Department of Nurse, Taoyuan Armed Forces General Hospital, Taoyuan 32549, Taiwan; 3 Division of Chest Medicine, Department of Internal Medicine, Cheng Hsin General Hospital, Taipei 11220, Taiwan; anemia0829@gmail.com; 4 Department of Medical Research, Tri-Service General Hospital, National Defense Medical Center, Taipei 11490, Taiwan; 5 School of Public Health, National Defense Medical Center, Taipei 11490, Taiwan; 6 Department of Emergency Medicine, Taoyuan Armed Forces General Hospital, Taoyuan 32549, Taiwan; 7 Department of Emergency Medicine, Tri-Service General Hospital, National Defense Medical Center, Taipei 11490, Taiwan; 8 Department of Neurology, Tri-Service General Hospital, National Defense Medical Center, Taipei 11490, Taiwan

**Keywords:** chronic obstructive pulmonary disease, atrial fibrillation, ischemic stroke, risk

## Abstract

Background: Atrial fibrillation (AF) and ischemic stroke (IS) are intricately linked to chronic obstructive pulmonary disease (COPD). Patients who suffer from both COPD and AF demonstrate a 2.85-fold greater risk of IS. However, the long-term risk remains insufficiently explored. Methods: This study utilized data from the Taiwanese National Health Insurance dataset spanning 2000 to 2015. Patients who were newly diagnosed with COPD, identified using the International Classification of Disease, Ninth Revision, Clinical Modification [ICD-9-CM] codes of 491, 492, and 496 and diagnosed with AF (ICD-9-CM code 427.3), were included in the study. The measured events included ISs (ICD-9-CM codes 433–437). Multivariate Cox proportional hazard models were employed to evaluate IS risk factors in this longitudinal analysis. Results: The combined presence of COPD and AF increased the risk of IS, with an adjusted hazard ratio of 5.722 (95% CI: 2.737–8.856, *p* < 0.001), AF without COPD with an adjusted HR of 3.506 (95% CI: 1.459–5.977, *p* < 0.001), and COPD with AF with an adjusted HR of 2.215 (95% CI: 1.099–3.538, *p* < 0.001) compared with patients without COPD and AF. Elderly patients exhibited a greater burden of cardiovascular comorbidities, including obstructive sleep apnea, thus further compounding the risk of IS. Conclusions: The coexistence of COPD and AF was associated with a markedly elevated risk of IS. The result highlights the additive and synergistic contributions of COPD and AF to the risk for IS. Aggressive treatment may mitigate the risk of IS.

## 1. Introduction

Chronic obstructive pulmonary disease (COPD) is the third leading cause of mortality worldwide, with an increasing incidence and prevalence being observed [1]. COPD is characterized by persistent respiratory symptoms with airflow limitations that result from airway and alveolar abnormalities; additionally, this condition is usually accompanied by a systemic inflammatory syndrome that affects other organs of the body. Current smoking, advanced age, and exposure to air pollution may induce COPD [2]. Moreover, COPD has been implicated in effects such as increased systemic inflammation, arterial stiffness, platelet activation, endothelial dysfunction, and hypoxia-induced oxidative stress, all of which contribute to an increased risk of cerebrovascular events [3].

Stroke may cause disability and mortality; additionally, a previous registry study noted that ischemic stroke (IS), which accounts for 70% of stroke cases, is the main type of stroke observed in Taiwan [4]. The prevalence of IS in patients with COPD has been reported to be 7.2% in Lithuania [5]. Moreover, a cross-sectional study demonstrated that the risk of IS was markedly elevated in patients with concomitant COPD and atrial fibrillation (AF), with an odds ratio (OR) of 2.85. However, this increased risk was not observed in individuals with AF alone or COPD alone [6]. In Sweden, COPD patients exhibited a 1.2-fold higher hazard ratio (HR) for all-cause stroke compared to controls, with the risk reaching a maximum value (HR 1.5) during the first two years following a COPD diagnosis [7]. The rate of stroke in the youngest age groups was observed to be higher than that in the older age groups with COPD. Furthermore, AF affects approximately 3% of adults and is a common heart disease [8].

Patients with COPD exhibit a 1.3-fold greater AF risk than COPD-free patients, which can increase by 2-fold during frequent exacerbations [9]. Additionally, the presence of new-onset AF in COPD patients was found to be associated with a 1.75-fold increase in IS risk over one year [10]. COPD is a preventable and treatable disease. In addition, the prevention of COPD and AF is essential for reducing the risk of IS, and healthcare providers should pay special attention to this risk [11].

Approximately half of the individuals with COPD exhibit more than three comorbidities, with the most common comorbidity being cardiovascular diseases. The coexistence of COPD and multiple comorbid conditions complicates the delineation of independent risk factors for stroke, as the potential confounding effects of COPD remain uncertain. Notably, COPD and stroke share several overlapping risk factors, thus leading to challenges in ascertaining whether the association between these conditions reflects direct causality or shared pathophysiology. Further studies are warranted to adjust for confounding variables and elucidate the precise relationship between COPD and stroke. Furthermore, IS patients with COPD have demonstrated reduced long-term survival probabilities [12].

Patients with COPD exhibit induced AF or IS, regardless of whether patients diagnosed with COPD and AF demonstrate a greater risk of IS. An understanding of these mechanisms underscores the importance of targeted interventions to reduce the burden of IS in this patient population. Therefore, we used the Taiwanese National Health Insurance Research Dataset to survey the relationships among COPD, AF, and IS by comparing patients in non-COPD and non-AF groups in order to determine the potential relationship between IS and individual or combined COPD and AF.

This study aimed to evaluate the individual risk of IS in patients with COPD and/or AF compared with COPD-free and AF-free patients. By investigating the risk of IS in relation to COPD and AF in a population observation study, this research aims to inform doctors and encourage more aggressive strategies for mitigating hypoxia and cardiac arrhythmia, thereby ultimately reducing stroke risk.

## 2. Materials and Methods

The National Health Insurance Dataset (NHIRD) provides comprehensive data, including date of birth, visit date, age, comorbid conditions, and codes for conditions such as COPD, AF and IS, as these records are required for healthcare providers to claim insurance payments by uploading these data. This study employed a retrospective cohort design and utilized data that were collected from the Longitudinal Taiwanese National Health Insurance Dataset from 1 January 2000 to 31 December 2015 [13].

This study analyzed both outpatient and inpatient datasets, thereby incorporating up to 3 diagnostic codes in the outpatient dataset and 5 diagnostic codes in the inpatient dataset. The payment data with respect to the insurance were obtained from the International Classification of Diseases, 9th Revision, Clinical Modification (ICD-9-CM) before 2016. In 2016, the Department of Taiwanese Center National Health Insurance began to use the 10th clinical revision of the International Classification of Diseases (ICD-10-CM).

**Figure 1:**
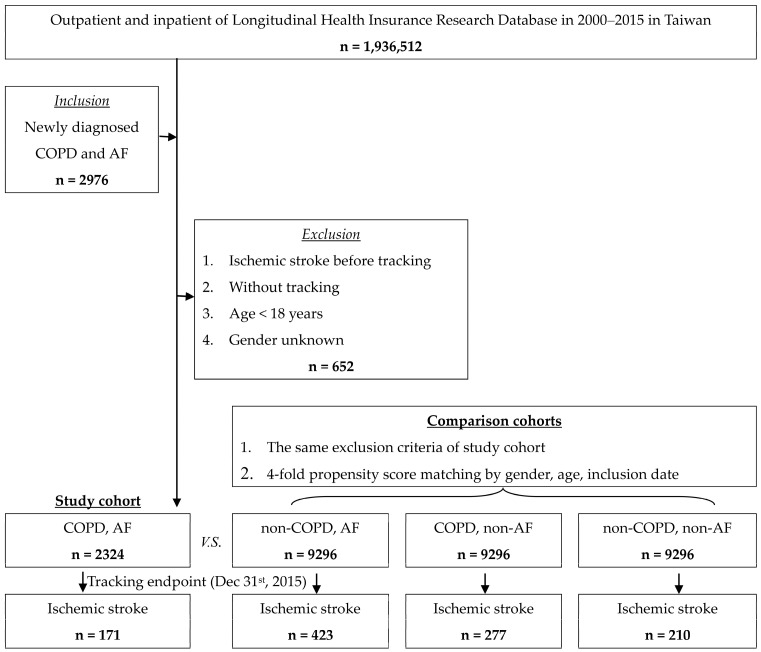
The flowchart of this study.

Adults who were aged 18 years or older with concurrent diagnoses of both COPD plus AF were included in the study as the index date, and the study period spanned January 2000–December 2015. COPD diagnosis was defined by ICD-9-CM codes 491, 493 and 496. AF diagnosis was identified using ICD-9-CM code 427.3. IS events were defined as outcomes which were classified by ICD-9-CM codes 433–437. The exclusion criteria included prior IS, indeterminate sex, and age younger than 18 years. The comparison group consisted of individuals who were matched 4:1 by age, sex, and index date and who did not have diagnoses of COPD and AF. Additionally, data were extracted for patients who were diagnosed with only COPD or only AF. The flowchart is shown in Figure 1.

The comorbidities including hypertension (401–405), diabetes mellitus (250), hyperlipidemia (272), coronary heart disease (410–414), congestive heart failure (428), sepsis (038, 003.1 and 036.1), chronic kidney disease (580–589), peripheral artery disease (443), anemia (285), alcohol related disorder (291,303, 305.1, and 571.0–571.4), hyperthyroidism (242), obstructive sleep apnea (327.23, 780.51, 780.53, and 780.57), autoimmune disorders (710), and rheumatoid arthritis (714) were mapped by using ICD-9-CM codes. Anatomical therapeutic chemical (ATC) classification codes were used to classify various drugs in this study, including ATC codes of long-acting muscarinic antagonists (LAMAs) (R03BB04, R03BB05, R03BB06 and R03BB07); ATC codes of long-acting β-agonists (LABAs) (R03AC12, R03AC13, R03AC16, R03AC18 and R03AC19); ATC codes of inhalation cortisone (ICS) (R03BA); ATC codes of LABA + ICS (R03AK06, R03AK07, R03AK08, R03AK09, R03AK10, R03AK11, R03AK12 and R03AK14); ATC codes of LAMA + LABA + ICS (R03AL08 and R03AL09); ATC codes of LABA + LAMA (R03AL03, R03AL04, R03AL05, R03AL06, R03AL07 and R03AL08); ATC codes of short-acting β-agonists (SABAs) (R03AC); and ATC codes of short-acting muscarinic antagonists (SAMAs) (R03BB01).

The chi-square test (X^2^) was used to evaluate the categorical factors, and one-way ANOVA was used for analyzing continuous variables with respect to differences. The risk factors for IS were analyzed with a multivariate Cox regression model. Moreover, *p* < 0.05 indicated statistical significance. SPSS software version 21 was used for the statistical analyses (International Business Machines Company, Armonk, NY, USA).

## 3. Results

**Figure 2:**
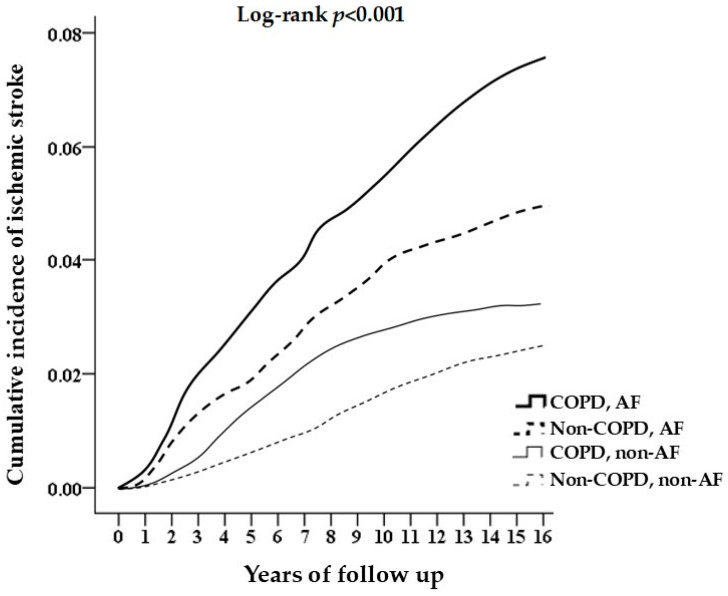
The cumulative incidence of ischemic stroke in different groups.

There were 171 ISs (7.36%) identified in patients with both COPD and AF, 423 ISs (4.55%) identified in patients with AF and without COPD, 277 ISs (2.98%) identified in COPD patients without AF, and 210 ISs (2.26%) identified in patients without COPD or AF during a mean 8.08 ± 5.9-year follow-up (Figure 2).

Greater proportions of coronary artery disease and congestive heart failure were observed in patients with COPD and AF, as well as in patients with AF without COPD. Additionally, the prevalence of autoimmune disease and rheumatic arthritis was notably greater in patients with both COPD and AF (Table 1).

The highest adjusted HR for IS was observed for IS in patients with both COPD and AF at 5.722 (95% CI: 2.737–8.856, *p* < 0.001), compared with patients without COPD and AF. The patients with AF but without COPD had an adjusted HR of 3.506 (95% CI: 1.459–5.977, *p* < 0.001). Those with COPD but without AF had an adjusted HR of 2.215 (95% CI: 1.099–3.538, *p* < 0.001). Adjusted HRs were also reported for other variables, including male sex (HR: 1.276; 95% CI: 1.017–1.506), age (HR: 1.498; 95% CI: 1.201–1.733), hypertension (HR: 2.165; 95% CI: 1.403–3.096), diabetes mellitus (HR: 1.522; 95% CI: 1.018–1.837), coronary artery disease (HR: 2.874; 95% CI: 1.73–3.539), chronic kidney disease (HR: 2.411; 95% CI: 1.395–3.106), peripheral artery occlusive disease (HR: 2.03; 95% CI: 1.304–2.798), obstructive sleep apnea (HR: 1.511; 95% CI: 1.014–1.986), and treatment with LAMA + LABA + ICS (HR: 1.42; 95% CI: 1.05–1.761, *p* = 0.025 compared with LAMA) (Table 2).

## 4. Discussion

A greater incidence of IS was observed in patients with both COPD and AF at the eight-year follow-up, thus highlighting a cumulative effect of IS in individuals with either condition. The HR associated with AF was greater than that associated with COPD, thereby indicating a stronger risk effect of AF. When considering the IS probability stratified by COPD and AF, we observed the highest risk in patients with COPD combined with AF, followed by patients with only AF and patients with only COPD compared with non-COPD and non-AF patients. Notably, the combination of COPD and AF further increased the risk of IS.

COPD has been identified as a risk factor for both AF [9] and stroke in a previous study [11]. Although COPD has not been listed as one of the risk factors for stroke by the American Stroke Association, emerging evidence has linked the two conditions. Patients with COPD have been shown to exhibit a 1.3-fold greater risk of experiencing a stroke than COPD-free patients in a previous meta-analysis [14]. IS is predominantly caused by atherosclerosis, which has established risk factors including hypertension, smoking, obesity, unhealthy dietary patterns, physical inactivity, dysregulated glycemic control, lipid abnormalities, alcohol consumption, psychosocial stressors, and cardiovascular diseases [15]. The key factors that promote pathophysiological changes in the context of COPD include systemic inflammation, hypoxia, hypercapnia, oxidative stress, and heightened sympathetic activation (11). Patients with COPD who are suffering from chronic, low-grade systemic inflammation may be susceptible to stroke development. Moreover, patients with COPD can suffer from increased hypoxia and reactive oxidative stress, and decreased antioxidant activity may be related to both IS and hemorrhagic stroke [11]. Results have shown that the RR was 1.11 for each 10% decrease in forced expiratory volume in one-second. Additionally, COPD has been shown to be associated with increased arterial stiffness, with small pulmonary vascular alterations resulting in atherosclerosis in COPD patients [16]. Acute exacerbations of COPD are particularly concerning, as they are associated with a twofold increased risk of IS within four days; however, no significant differences have been demonstrated from a chronic perspective in previous studies [17].

Patients with COPD had a higher prevalence of AF. A previous study demonstrated that patients with both AF and COPD had an OR of 2.9 for IS compared with patients with AF or COPD alone [6]. Moreover, previous results have shown that there was a greater risk of AF in COPD patients with advanced age, male sex, atherosclerosis factors, obstructive sleep apnea, thyroid dysfunction, and overweight, smoking and drinking statuses [18]. Hypoxia with ventilation limitations in COPD may induce hyperventilation with sympathetic hyperactivity, prolonged QTc intervals, and right ventricular hypertrophy by hypercapnia and acidosis, thereby resulting in pulmonary hypertension with right atrial dilatation [19]. AF is also an essential cardiac-related factor. The treatment of COPD with beta agonists shortens the atrial unresponsiveness time; additionally, anticholinergic agents increase sympathetic activity, and methylxanthines and corticosteroids cause the excretion of potassium [20]. Patients with COPD who are treated with SABAs and both LABA + LAMA may demonstrate increased cardiac arrhythmia risks with potential stroke risks. The risk of cardiac arrhythmia in SABAs has demonstrated an RR of 1.27, and that in LAMAs has demonstrated an RR of 1.47 [21]. Furthermore, the patients with COPD who received inhaled LABAs (with an HR of 2.38), SABAs (with an HR of 2.02), and ICS/LABAs (with an HR of 2.08) were demonstrated to be at greater risk of major adverse cardiovascular events than those receiving SAMA treatment [20].

The cause of AF in COPD patients is related to elevated plasma high-sensitivity chronic reaction proteins and interleukin 6, which are associated with an increased AF risk [9]. Thrombocytosis has been noted in patients with COPD, and the mean platelet volume has been shown to be increased during the stable phase [22]. Fibrin clots in patients with COPD are resistant to lysis in the proinflammatory state. Moreover, chronic reaction protein levels were decreased when patients were treated with corticosteroids. In COPD patients, the use of SABAs is associated with an increased risk of stroke (with an HR of 1.7), and combination treatment with LABAs and ICSs is related to a risk reduction (with an HR of 0.8) within 3 years [23]. Inhaled ipratropium bromide is also associated with stroke risk in COPD patients, and this effect depends on the duration of treatment and combination with SABA or theophylline [24]. Our study revealed that patients with COPD who received LAMA + LABA + ICS had increased IS compared with patients who received LAMA only. The potential reason was that the administration of multiple medications increased the risk of arrhythmia.

The incidence of IS was greater in male patients with COPD than in male patients without COPD (with an IRR of 1.04); however, there was no difference in females. The incidence of IS is double in male patients with COPD compared to female patients with COPD (with an IRR of 2) [25]. Our study revealed a 1.3-fold increased risk of IS. More attention should be provided to male patients with respect to suitable treatment and education. The IS risk of hypertension has been shown to be 2.37, with that of diabetes being 1.60, and that of cardiac causes being 2.74, based on multicountry studies [15]. Poor control in diabetic patients may induce polyneuropathy with hypersympathetic activity accompanied by AF and vascular injury. Similarly, our study revealed that the IS risk associated with hypertension was 2.2-fold greater, the IS risk associated with diabetes mellitus was 1.5-fold greater, the IS risk associated with coronary artery disease was 2.9-fold greater, and the IS risk associated with AF was 3.5-fold greater. In a previous study, peripheral artery involvement was demonstrated to cause 0.87 cases/year of IS and TIA, with a reduced risk being observed after antiplatelet therapy [26]. Our study revealed a 2-fold increased IS risk in patients with peripheral artery disease. Moreover, OSA is associated with a risk of new-onset AF occurrence (with an HR of 2.88); additionally, an HR of 5.84 has been observed for the risk of stroke events in obese patients [27]. Our study also demonstrated a risk of IS.

Hyperlipidemia is associated with a 2-fold increase in IS risk in patients with COPD [10]. However, in this study, the patients with hyperlipidemia did not exhibit an IS risk because they received lipid-lowering treatments for related diagnostic codes in the claims data. The IS risks of autoimmune disease and rheumatic arthritis did not significantly increase because the patients who were receiving immune therapy exhibited reduced inflammation according to the claims data. Moreover, a higher risk of stroke was observed with embolization and hypoperfusion with right heart failure in patients with COPD [28]. Patients with congestive heart failure demonstrate more severe conditions with a higher mortality rate than those with reduced life expectancies without significant IS risks. Furthermore, a previous study observed that the risk of AF after sepsis was 1.8 within 1 year [29]. In this study, there was a lower prevalence of sepsis (approximately 1.46%) which was associated with no significant IS risk.

Physicians in the fields of chest medicine and neurology should focus increased attention on the preventive management of COPD to reduce the risks of stroke. Previous results have shown that older females with COPD who received more than four influenza vaccine injections exhibited a 0.3-fold greater risk of stroke in Taiwan [30]. Additionally, in patients with COPD and a greater risk of IS, regular vaccination is beneficial for reducing stroke. Moreover, aspirin use has been shown to be associated with a lower risk of IS in patients with COPD, with an IRR of 1.28 compared to an IRR of 1.63 without treatment [17]. The administration of antiplatelet treatment is also correlated with a reduction in mortality in COPD patients [31]. Furthermore, statins appear to have beneficial effects on COPD, and it has been suggested that they may reduce the risks of exacerbation [32] and mortality [33].

This study provides insights into COPD and AF burden and subsequent IS incidence. We further considered the possibility that IS with preconditioning in COPD and AF patients may affect IS occurrence to different degrees. COPD-induced AF or IS was clearly established in a previous study, and our study confirmed an amplified effect. We estimated that the IS risk of both COPD and AF was greater than that of any individual factor related to IS in the clinical setting.

There were several limitations in this study. First, the diagnosis of COPD lacks an objective method and is potentially overestimated by using the ICD-9-CM. A previous study reported that only 2.65% of patients with COPD used spirometry for diagnosis, and the majority of COPD patients used the ICD-9/10-CM classification method [6]. Fewer spirometry procedures being performed may lead to less information on COPD severity. Second, there was a lack of information regarding smoking, drinking and exercise activities in the claim dataset. The consumptions of tobacco and alcohol were shown to increase the risk of IS in a previous study [15]. Smoking patients easily develop COPD and drinking patients frequently develop alcohol related disorder. We attempted to reduce confounding factors related to smoking and drinking, whereby we adjusted for COPD and alcohol related disorder. Registered studies need to address this possible methodological gap. Third, although our IS incidence studied at approximately 8 years in the Chinese population, other ethnic groups need to be evaluated. Fourth, the control group was matched for age, sex, and inclusion date with patients with COPD and AF in our study, although propensity score matching was not performed. This study revealed an increased incidence of coronary artery disease and congestive heart failure in patients with COPD and AF, but we attempted to reduce interference with the risk of IS by adjusting for coronary artery disease and congestive heart failure. Finally, the risk of IS was greater in patients with COPD after severe exacerbation than in those after moderate exacerbation [17]; a future registered study is needed to confirm this result.

## 5. Conclusions

This is the first study demonstrating a significantly increased long-term risk of IS in patients with combined COPD and AF in a Chinese population. Patients with both COPD and AF exhibited a nearly 6-fold increased risk of IS. Furthermore, the presence of obstructive sleep apnea further compounded the risk of IS. Patients with COPD must be aware of this risk, which is beneficial for both the effective treatment of COPD combined with AF and the implementation of appropriate preventive strategies against embolic IS.

## Figures and Tables

**Table 1:** The baseline characteristics of patients in this study.

Variables	Total 30,212	COPD, AF 2324	non-COPD, AF 9296	COPD, non-AF 9296	Non-COPD, Non-AF 9296	*p*
Sex (male)	15,561 (51.50%)	1197 (51.51%)	4788 (51.51%)	4788 (51.51%)	4788 (51.51%)	0.999
Age (years)	43.25 ± 18.41	43.26 ± 18.45	43.23 ± 18.39	43.21 ± 18.33	43.29 ± 18.51	0.87
Hypertension	6235 (20.64%)	520 (22.38%)	1906 (20.5%)	1913 (20.58%)	1896 (20.4%)	0.192
Diabetes mellitus	7100 (23.5%)	567 (24.4%)	2203 (23.7%)	2210 (23.77%)	2120 (22.81%)	0.248
Hyperlipidemia	4991 (16.52%)	422 (18.16%)	1537 (16.53%)	1529 (16.45%)	1503 (16.17%)	0.145
Coronary artery disease	4316 (14.29%)	382 (16.43%)	1422 (15.3%)	1267 (13.63%)	1245 (13.39%)	<0.001 *
Congestive heart failure	1562 (5.17%)	148 (6.37%)	511 (5.5%)	459 (4.94%)	444 (4.78%)	0.005 *
Sepsis	433 (1.43%)	35 (1.51%)	136 (1.46%)	134 (1.44%)	128 (1.38%)	0.949
Chronic kidney disease	4945 (16.37%)	388 (16.70%)	1539 (16.56%)	1562 (16.8%)	1456 (15.66%)	0.166
Peripheral artery disease	2749 (9.1%)	232 (9.98%)	888 (9.55%)	823 (8.85%)	806 (8.67%)	0.064
Anemia	390 (1.29%)	28 (1.2%)	124 (1.33%)	118 (1.27%)	120 (1.29%)	0.959
Alcohol related disorder	1550 (5.13%)	126 (5.42%)	483 (5.2%)	476 (5.12%)	465 (5%)	0.849
Hyperthyroidism	407 (1.35%)	30 (1.29%)	128 (1.38%)	131 (1.41%)	118 (1.27%)	0.847
Obstructive sleep apnea	2642 (8.74%)	211 (9.08%)	829 (8.92%)	813 (8.75%)	789 (8.49%)	0.695
Autoimmune disease	2125 (7.03%)	193 (8.3%)	678 (7.29%)	656 (7.06%)	598 (6.43%)	0.008 *
Rheumatic arthritis	1344 (4.45%)	140 (6.02%)	423 (4.55%)	402 (4.32%)	379 (4.08%)	0.001 *

* *p* < 0.05.

**Table 2:** Risk factors for ischemic stroke.

Variables	Adjusted Hazard Ratio (95% CI)	*p*
COPD plus AF	5.722 (2.737–8.856)	<0.001 *
AF without COPD	3.506 (1.459–5.977)	<0.001 *
COPD without AF	2.215 (1.099–3.538)	<0.001 *
COPD-free and AF-free	Reference	
Gender (male)	1.276 (1.017–1.506)	0.026 *
Age (years)	1.498 (1.201–1.733)	<0.001 *
Hypertension	2.165 (1.403–3.096)	<0.001 *
Diabetes mellitus	1.522 (1.018–1.837)	0.029 *
Hyperlipidemia	1.053 (0.812–1.532)	0.196
Coronary artery disease	2.874 (1.73–3.539)	<0.001 *
Congestive heart failure	1.03 (0.642–1.173)	0.395
Sepsis	1.126 (0.789–1.674)	0.329
Chronic kidney disease	2.411 (1.395–3.106)	<0.001 *
Peripheral artery disease	2.03 (1.304–2.798)	<0.001 *
Anemia	1 (0.364–1.853)	0.812
Alcohol related disorder	1.234 (0.824–1.598)	0.168
Hyperthyroidism	1.051 (0.783–1.41)	0.279
Obstructive sleep apnea	1.511 (1.014–1.986)	0.027 *
Autoimmune disease	1.203 (0.798–1.625)	0.202
Rheumatic arthritis	1.042 (0.633–1.431)	0.378
COPD treatment		
LAMA	Reference	
LABA	1.024 (0.676–1.579)	0.513
LAMA + ICS	1.164 (0.804–1.672)	0.467
LABA + ICS	1.152 (0.833–1.683)	0.421
LAMA + LABA + ICS	1.42 (1.05–1.761)	0.025 *
LAMA + LABA	1.206 (0.838–1.724)	0.378
SABA	1.003 (0.638–1.806)	0.335
SAMA	1.207 (0.527–1.43)	0.512
SABA + SAMA	1.015 (0.803–1.477)	0.137

* *p* < 0.05; COPD: chronic obstructive pulmonary disease; AF: atrial fibrillation; CI: confidence interval; LAMAs; long-acting muscarinic antagonists; LABAs: long-acting β-agonists; ICS: inhaled cortisone; SABAs: short-acting β-agonists; SAMAs: short-acting muscarinic antagonists.

## Data Availability

Restrictions apply to the availability of these data. Data were obtained from the National Health Insurance database and are available from the authors with the permission of the National Health Insurance Administration of Taiwan.

## References

[1] Chen S., Kuhn M., Prettner K., Yu F., Yang T., Bärnighausen T., Bloom D.E., Wang C. (2023). The global economic burden of chronic obstructive pulmonary disease for 204 countries and territories in 2020–50: A health-augmented macroeconomic modelling study. Lancet Glob. Health.

[2] Safiri S., Carson-Chahhoud K., Noori M., Nejadghaderi S.A., Sullman M.J.M., Ahmadian Heris J., Ansarin K., Mansournia M.A., Collins G.S., Kolahi A.A. (2022). Burden of chronic obstructive pulmonary disease and its attributable risk factors in 204 countries and territories, 1990–2019: Results from the Global Burden of Disease Study 2019. BMJ.

[3] Almagro P., Boixeda R., Diez-Manglano J., Gómez-Antúnez M., López-García F., Recio J. (2020). Insights into Chronic Obstructive Pulmonary Disease as Critical Risk Factor for Cardiovascular Disease. Int. J. Chronic Obstr. Pulm. Dis..

[4] Hsieh F.I., Lien L.M., Chen S.T., Bai C.H., Sun M.C., Tseng H.P., Chen Y.W., Chen C.H., Jeng J.S., Tsai S.Y. (2010). Get with the Guidelines-Stroke performance indicators: Surveillance of stroke care in the Taiwan Stroke Registry: Get with the Guidelines-Stroke in Taiwan. Circulation.

[5] Puteikis K., Mameniškienė R., Jurevičienė E. (2021). Neurological and Psychiatric Comorbidities in Chronic Obstructive Pulmonary Disease. Int. J. Chronic Obstr. Pulm. Dis..

[6] Nadeem R., Sharieff A., Tanna S., Sidhu H., Molnar J., Nadeem A. (2015). Potential Augmentation of the Risk of Ischemic Cerebrovascular Accident by Chronic Obstructive Pulmonary Disease in Patients with Atrial Fibrillation. J. Stroke Cerebrovasc. Dis..

[7] Söderholm M., Inghammar M., Hedblad B., Egesten A., Engström G. (2016). Incidence of stroke and stroke subtypes in chronic obstructive pulmonary disease. Eur. J. Epidemiol..

[8] Zulkifly H., Lip G.Y.H., Lane D.A. (2018). Epidemiology of atrial fibrillation. Int. J. Clin. Pract..

[9] Grymonprez M., Vakaet V., Kavousi M., Stricker B.H., Ikram M.A., Heeringa J., Franco O.H., Brusselle G.G., Lahousse L. (2019). Chronic obstructive pulmonary disease and the development of atrial fibrillation. Int. J. Cardiol..

[10] Liu C.C., Chen Y.H., Chang Y.H., Chien W.C., Lin H.C., Cheng C.G., Cheng C.A. (2022). New-Onset Atrial Fibrillation Is a Risk Factor of Ischemic Stroke in Chronic Obstructive Pulmonary Disease. Healthcare.

[11] Corlateanu A., Covantev S., Mathioudakis A.G., Botnaru V., Cazzola M., Siafakas N. (2018). Chronic Obstructive Pulmonary Disease and Stroke. COPD.

[12] Bavishi S., Chaudhary D., Li J., Naik S., Abedi V., Zand R. (2022). Long-term mortality in ischemic stroke patients with concomitant chronic obstructive pulmonary disease. J. Stroke Cerebrovasc. Dis..

[13] National Health Insurance Research Database. https://dep.mohw.gov.tw/DOS/lp-2506-113.html.

[14] Kim Y.R., Hwang I.C., Lee Y.J., Ham E.B., Park D.K., Kim S. (2018). Stroke risk among patients with chronic obstructive pulmonary disease: A systematic review and meta-analysis. Clinics.

[15] O’Donnell M.J., Xavier D., Liu L., Zhang H., Chin S.L., Rao-Melacini P., Rangarajan S., Islam S., Pais P., McQueen M.J. (2010). Risk factors for ischaemic and intracerebral haemorrhagic stroke in 22 countries (the INTERSTROKE study): A case-control study. Lancet.

[16] Roeder M., Sievi N.A., Kohlbrenner D., Clarenbach C.F., Kohler M. (2020). Arterial Stiffness Increases Over Time in Relation to Lung Diffusion Capacity: A Longitudinal Observation Study in COPD. Int. J. Chronic Obstr. Pulm. Dis..

[17] Rothnie K.J., Connell O., Müllerová H., Smeeth L., Pearce N., Douglas I., Quint J.K. (2018). Myocardial Infarction and Ischemic Stroke after Exacerbations of Chronic Obstructive Pulmonary Disease. Ann. Am. Thorac. Soc..

[18] Goudis C.A. (2017). Chronic obstructive pulmonary disease and atrial fibrillation: An unknown relationship. J. Cardiol..

[19] Goudis C.A., Konstantinidis A.K., Ntalas I.V., Korantzopoulos P. (2015). Electrocardiographic abnormalities and cardiac arrhythmias in chronic obstructive pulmonary disease. Int. J. Cardiol..

[20] Amegadzie J.E., Gamble J.M., Farrell J., Gao Z. (2022). Association between Inhaled β2-agonists Initiation and Risk of Major Adverse Cardiovascular Events: A Population-based Nested Case-Control Study. Int. J. Chronic Obstr. Pulm. Dis..

[21] Wilchesky M., Ernst P., Brophy J.M., Platt R.W., Suissa S. (2012). Bronchodilator use and the risk of arrhythmia in COPD: Part 2: Reassessment in the larger Quebec cohort. Chest.

[22] Fawzy A., Putcha N., Paulin L.M., Aaron C.P., Labaki W.W., Han M.K., Wise R.A., Kanner R.E., Bowler R.P., Barr R.G. (2018). Association of thrombocytosis with COPD morbidity: The SPIROMICS and COPDGene cohorts. Respir. Res..

[23] Lin H.W., Chung C.L., Lin Y.S., Yu C.M., Lee C.N., Bien M.Y. (2015). Inhaled Pharmacotherapy and Stroke Risk in Patients with Chronic Obstructive Pulmonary Disease: A Nationwide Population Based Study Using Two-Stage Approach. PLoS ONE.

[24] Wang M.T., Tsai C.L., Lo Y.W., Liou J.T., Lee W.J., Lai I.C. (2012). Risk of stroke associated with inhaled ipratropium bromide in chronic obstructive pulmonary disease: A population-based nested case-control study. Int. J. Cardiol..

[25] de Miguel-Díez J., López-de-Andrés A., Jiménez-García R., Hernández-Barrera V., Jiménez-Trujillo I., Ji Z., de Miguel-Yanes J.M., López-Herranz M. (2021). Sex Differences in the Incidence and Outcomes of COPD Patients Hospitalized with Ischemic Stroke in Spain: A Population-Based Observational Study. Int. J. Chronic Obstr. Pulm. Dis..

[26] Kolls B.J., Sapp S., Rockhold F.W., Jordan J.D., Dombrowski K.E., Fowkes F.G.R., Mahaffey K.W., Berger J.S., Katona B.G., Blomster J.I. (2019). Stroke in Patients with Peripheral Artery Disease. Stroke.

[27] Dalmar A., Singh M., Heis Z., Cumpian T.L., Ceretto C., Mortada M.E., Bhatia A., Niazi I., Chua T.Y., Sra J. (2021). Risk of Atrial Fibrillation and Stroke After Bariatric Surgery in Patients with Morbid Obesity with or Without Obstructive Sleep Apnea. Stroke.

[28] Orea-Tejeda A., Bozada-Gutiérrez K., Pineda-Juárez J., González-Islas D., Santellano-Juárez B., Keirns-Davies C., Peláez-Hernández V., Hernández-Zenteno R., Sánchez-Santillán R., Cintora-Martínez C. (2017). Right Heart Failure as a Risk for Stroke in Patients with Chronic Obstructive Pulmonary Disease: A Case-Control Study. J. Stroke Cerebrovasc. Dis..

[29] Cheng C.A., Cheng C.G., Lin H.C., Lee J.T., Lin H.C., Cheng C.C., Chien W.C., Chiu H.W. (2017). New-onset atrial fibrillation-related ischemic stroke occurring after hospital discharge in septicemia survivors. QJM.

[30] Chen C.C., Lin C.H., Chiu C.C., Yang T.Y., Hsu M.H., Wang Y.H., Lei M.H., Yeh H.T., Fang Y.A., Hao W.R. (2022). Influenza Vaccination and Risk of Stroke in Women with Chronic Obstructive Pulmonary Disease: A Nationwide, Population-Based, Propensity-Matched Cohort Study. Front. Med..

[31] Pavasini R., Biscaglia S., d’Ascenzo F., Del Franco A., Contoli M., Zaraket F., Guerra F., Ferrari R., Campo G. (2016). Antiplatelet Treatment Reduces All-Cause Mortality in COPD Patients: A Systematic Review and Meta-Analysis. COPD.

[32] Lin C.M., Yang T.M., Yang Y.H., Tsai Y.H., Lee C.P., Chen P.C., Chen W.C., Hsieh M.J. (2020). Statin Use and the Risk of Subsequent Hospitalized Exacerbations in COPD Patients with Frequent Exacerbations. Int. J. Chronic Obstr. Pulm. Dis..

[33] Horita N., Miyazawa N., Kojima R., Inoue M., Ishigatsubo Y., Ueda A., Kaneko T. (2014). Statins reduce all-cause mortality in chronic obstructive pulmonary disease: A systematic review and meta-analysis of observational studies. Respir. Res..

